# Modification of cellular membranes conveys cryoprotection to cells during rapid, non-equilibrium cryopreservation

**DOI:** 10.1371/journal.pone.0205520

**Published:** 2018-10-10

**Authors:** Jan Huebinger

**Affiliations:** Department of Systemic Cell Biology, Max Planck Institute of Molecular Physiology, Dortmund, Germany; Hungarian Academy of Sciences, HUNGARY

## Abstract

Rapid cooling and re-warming has been shown promising to cryopreserve living cells, which cannot be preserved by conventional slow freezing methods. However, success is limited by the cytotoxicity of highly concentrated cryoprotective agents. Recent results have shown that cryoprotective agents do not need to suppress intracellular ice crystals completely to allow for survival after cryopreservation. Cryoprotective agents like DMSO or ethylene glycol can also lead to a tolerance of cells towards intracellular ice. It is however unclear by which mechanism this tolerance is achieved. These substances are also known to modulate properties of cellular membranes. It is shown here that cryoprotective DMSO and ethylene glycol have a clear influence on the mobility of lipids in the plasma membrane of HeLa cells. To isolate changes of the properties of plasma membranes from effects on ice formation, the membrane properties were modulated in absence of cryoprotective agents. This was achieved by changing their sterol content. In cells with elevated sterol content, an immobile lipid fraction was present, similar to cells treated with DMSO and ethylene glycol. These cells showed also significantly increased plasma membrane integrity after rapid freezing and thawing in the absence of classical cryoprotective agents. However, their intracellular lysosomes, which cannot be enriched with sterols, still got ruptured. These results clearly indicate that a modulation of membrane properties can convey cryoprotection. Upon slow cooling, elevated sterol content had actually an adverse effect on the plasma membranes, which shows that this effect is specific for rapid, non-equilibrium cooling processes. Unraveling this alternative mode of action of cryoprotection should help in the directed design of novel cryoprotective agents, which might be less cytotoxic than classical, empirically-found cryoprotective agents.

## Introduction

Cryopreservation, i.e. the potentially infinite storage under very cold temperatures, of living cells is of fundamental interest for biomedical research, clinical application and the preservation of endangered species. Classical slow cooling cryopreservation works by extracting water from the cells and thereby constraining ice crystallization to the extracellular medium [1]. This is accompanied by a massive shrinkage of the cells and success of reversibility depends on energy demanding adaptation by the cells [2]. Immortalized laboratory cell lines are usually well adapted to this, but many other cell types do not tolerate this. Therefore, rapid cooling and re-warming (often termed vitrification) is a very promising approach for the cryopreservation of cells that cannot be efficiently preserved by slow cooling approaches (e.g. [3,4]). However, this approach suffers from toxicity of the relatively high concentrated cryoprotective agents that need to be applied to the cells at temperatures above 0°C [1,5]. These cryoprotecants were thought to be necessary to avoid ice-crystallization in cells, since ice-crystals were–in analogy to slow freezing approaches–considered to be absolutely lethal [1,5]. However, in a recent study we showed that ice-crystals actually form during some of these applications, which nevertheless allowed for very high survival rates [6]. Based on this, the term vitrification is not strictly correct for such applications, because it would imply the complete suppression of ice crystallization. These approaches are therefore called rapid-cooling and rewarming approaches here. Using such approaches, the total amount of ice or the number of ice crystals did not correlate with an increase of cell death, demonstrating that intracellular ice crystallization is not lethal upon fast cooling and warming. However, cell death occurred when samples were slowly warmed and ice could re-crystallize to fewer but bigger ice-crystals [6]. This correlation does not prove causality between re-crystallization and cell death. Yet, it reopens the question of the cause of cell death and with that also the mode of action of cryoprotective agents. The amount of tolerable re-crystallization is dependent on the type of cryoprotective agents used [6]. This clearly indicates that the cryoprotective effect is not solely prevention of ice nucleation or re-crystallization. The cryoprotective agents apparently provide protection against the harmful effects, which at least coincide with re-crystallization. The two most frequently considered types of cryodamage are direct damage by ice crystals to cellular membranes and high solute concentration in the unfrozen fraction around the ice crystals, which could lead to the denaturing of cellular proteins or damage to lipid bilayer membranes [1,7]. However, lipid bilayer membranes themselves also undergo phase transitions and structural changes upon cooling [8,9], which have been associated with cold shock damage in sperm cells [10]. In all of these cases, membranes are a target for cryodamage. Small polar molecules like DMSO, glycerol or ethylene glycol can modulate the hydration layer of membranes [11], which changes their properties at subzero temperatures [8,12]. These substances generate also a high tolerance against re-crystallization after rapid cooling [6]. It is therefore conceivable that they convey cryoprotection to the membranes under cryo conditions. Here, I tested therefore, if these cryoprotective agents change membrane properties and if membranes can be cryoprotected by modulating only their properties, i.e. without inhibition of ice crystallization. Modulation of plasma membranes by increasing sterol content, resulted in a clear increase in resistance to cryodamage. Lysosomes, which cannot be enriched with sterols [13], were still found ruptured. Additionally, fluorescence recovery after photobleaching (FRAP) analysis in living HeLa cells, showed a similar partitioning effect on the plasma membrane by cryoprotective agents and sterol enrichment. On the other hand, a marked denaturing of cytosolic proteins–as it has been observed upon relatively slow and lethal cooling [9]–was not detectable by circular dichroism spectroscopy after lethal rapid cooling and rewarming. The observed improved resistance to cryodamage by increasing sterol content was specific for rapid cooling processes.

These results thus show that cryodamage during rapid, non-equilibrium cooling processes involves a damage to cellular membranes that can be prevented by modulating only membrane properties in the absence of cryoprotectants, which would reduce ice formation. The results further imply that this conveys at least part of the cryoprotective effect of small polar substances like DMSO or ethylene glycol.

## Results

**Fig 1:**
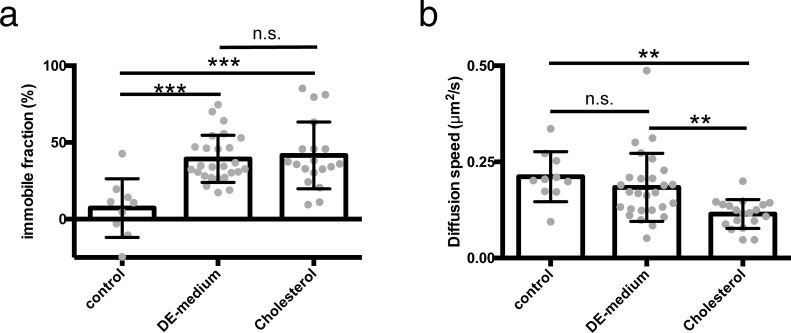
FRAP measurements of DiOC18 in the plasma membrane of HeLa cells treated with DMSO and ethylene glycol or enriched with cholesterol. HeLa cells have been labeled with DiOC18. They have been kept in phosphate buffered medium (control), treated with a combination of 15% DMSO and 15% ethylene glycol (DE-medium) or enriched with cholesterol by treating them with 10 mM cholesterol-loaded methyl-β-cyclodextrin for 60 min (Cholesterol). A section of the plasma membrane (app. 2 μm) has been bleached and the fluorescence recovery has been recorded for 100 s. From this data, the immobile fraction (a) and the diffusion speed of the mobile fraction (b) have been calculated. Data is shown as single cell measurements (gray dots) and mean +/- s.d. (black bars); n = 10 (control); n = 22 (DE-medium); n = 19 (Cholesterol); ***: p<0.001; **:p<0.01 using parametric one-way ANOVA and Tukey post-hoc test.

To gain understanding about the effect that a medium of 15% DMSO and 15% ethylene glycol (DE-medium) has on membranes of living cells, living HeLa cells were labeled with the fluorescent lipid DiOC18 and treated with DE-medium. Then a small section of the plasma membrane of these cells was bleached to perform fluorescence recovery after photobleaching (FRAP) experiments. In cells treated with DE-medium the fluorescence did not fully recover, indicating the presence of an (on the timescale of the experiment) immobile phase within the plasma membrane, which was not present in untreated cells (Fig 1A). The diffusion speed in the mobile fraction was however unaltered (Fig 1B).

**Fig 2:**
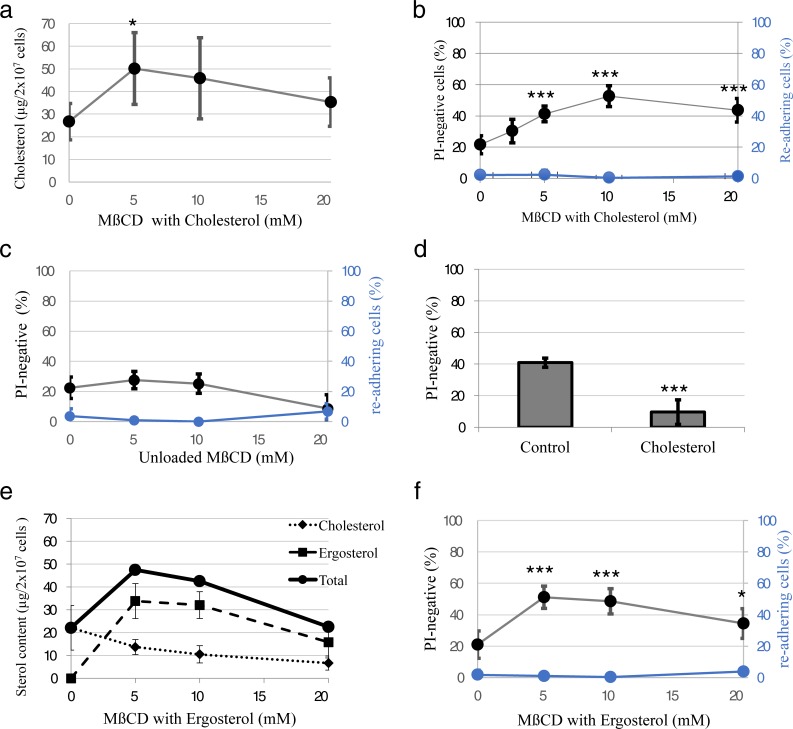
Effects of sterol-loading on HeLa cells after cryopreservation. **a)** HeLa cells have been enriched with cholesterol by treating them with 0–20 mM methyl-β-cyclodextrin (MβCD) with cholesterol for 60 min. Afterwards their cholesterol content has been measured fluorometrically using enzyme-coupled reactions to produce fluorescent resorufin. n = 3; *: p<0.05 vs 0 mM using student´s t-test **b)** HeLa cells treated with 0–20 mM MβCD with cholesterol for 60 min have been rapidly frozen without cryoprotectants in open pulled straws (OPS) in liquid nitrogen. After thawing in cell culture medium at 37°C, the plasma membrane integrity of the cells has been tested by propidium iodide (PI) staining (black circles; left y-axis; n = 4–6) and the ability of cells to re-adhere has been tested (blue circles; right y-axis; n = 12). ***: p<0.001 vs 0 mM using student´s t-test **c)** HeLa cells treated with 0–20 mM unloaded MβCD for 60 min have been rapidly frozen without cryoprotectants in OPS in liquid nitrogen. After thawing in cell culture medium at 37°C, the plasma membrane integrity of the cells has been tested by propidium iodide (PI) staining (black circles; left y-axis; n = 4–6) and the ability of cells to re-adhere has been tested (blue circles; right y-axis; n = 3–5). **d)** HeLa cells treated with 10 mM MβCD with cholesterol for 60 min (Cholesterol) and untreated control cells have been slowly frozen without cryoprotectants with 1°C/min. After thawing in cell culture medium at 37°C, the plasma membrane integrity of the cells has been tested by PI staining. n = 6; ***: p<0.001 vs control using student´s t-test **e)** HeLa cells have been enriched with ergosterol by treating them with 0–20 mM ergosterol-loaded MβCD for 60 min. Afterwards their cholesterol content has been measured fluorometrically using enzyme-coupled reactions to produce fluorescent resorufin and their ergosterol content has been measured spectroscopically. Total sterol content is depicted as the sum of the mean values of ergosterol and cholesterol content. n = 3 **f)** HeLa cells treated with 0–20 mM MβCD with ergosterol for 60 min have been rapidly frozen without cryoprotectants in OPS in liquid nitrogen. After thawing in cell culture medium at 37°C, the plasma membrane integrity of the cells has been tested by propidium iodide (PI) staining (black circles; left y-axis; n = 4–6) and the ability of cells to re-adhere has been tested (blue circles; right y-axis; n = 3–4). *: p<0.05, ***: p<0.001 vs 0 mM using student´s t-test.

To modulate plasma membrane properties of living HeLa cells, their cholesterol content was altered. Treatment of the cells with 5–20 mM cholesterol-loaded methyl-β-cyclodextrin (MβCD) for 60 min, led to an increased cellular cholesterol content (Fig 2A). The plasma membrane of HeLa cells treated with 10 mM of cholesterol in medium without cryoprotective agents showed in FRAP experiments an overall decrease in diffusion speed (Fig 1B) and a similar increase in immobile lipids as cells with normal cholesterol content in DE-medium (Fig 1A).

To test the integrity of plasma membranes upon relatively rapid cooling and warming, we cooled cells in open pulled straws (OPS; [14]) in liquid nitrogen and subsequently rewarmed them in 37°C-warm cell culture medium. Cooling cells in OPS without cryoprotective agents leads to formation of hexagonal ice crystals and is lethal to the cells [6]. Membrane integrity was tested by propidium iodide (PI) staining after rewarming. The membrane-impermeable PI selectively stains cells with compromised plasma membrane integrity by strong red fluorescence after intercalating with their DNA. In HeLa cells with normal cholesterol content rapidly cooled and rewarmed in the absence of cryoprotective agents, a fraction of 21.5 +/- 5.7% (mean +/- s.d.) had intact plasma membranes shortly after rewarming. By increasing the cholesterol content, this was significantly increased to a maximum of 52.7 +/- 6.7% (mean +/- s.d.; p<0.001 using student’s t-test; Fig 2B), demonstrating a much better resilience of these membranes against cryodamage. A treatment with MβCD alone, which extracts Cholesterol from the cells, did not lead to a significant increase in plasma membrane integrity (Fig 2C). This demonstrates that there is no cryoprotective effect by MβCD itself and decreasing the cholesterol content has no cryoprotective effect on the plasma membranes. When cells were slow-frozen with 1°C/min, increasing the sterol content actually has a detrimental effect on plasma membrane integrity (Fig 2D). Treating cells with MβCD loaded with ergosterol–which led to an increase of total sterol content to a very similar extent as cholesterol, but a decrease in cholesterol (Fig 2E)–yielded in very similar results as the treatment with cholesterol-loaded MβCD (Fig 2F). Together this demonstrates a cryoprotective effect on the plasma membranes by a modulation of their properties only.

**Fig 3:**
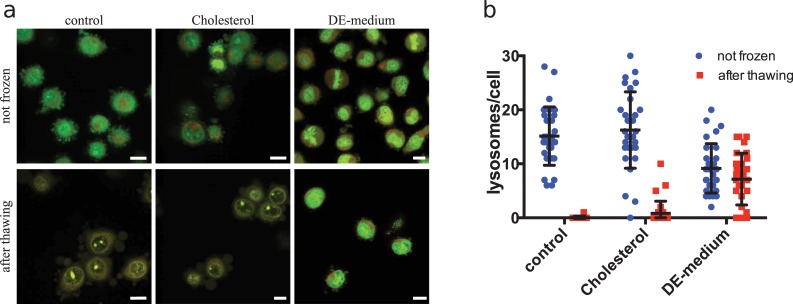
Staining of lysosomes in HeLa cells treated with DMSO and ethylene glycol or enriched with cholesterol after rapid freezing and thawing. HeLa cells were labeled with acridine orange and kept in phosphate buffer (control), treated with a combination of 15% DMSO and 15% ethylene glycol (DE-medium) or enriched with cholesterol by treatment with 10 mM cholesterol-loaded methyl-β-cyclodextrin for 60 min (Cholesterol). Cells were imaged on a confocal microscope using the 488-nm laser line of an argon laser. Green (500–560 nm) and red (600–700 nm) emission light was collected in separate channels. Lysosomes appear as dots in the red channel. In **a**) representative overlay images of green and red fluorescence are shown. The experiment was performed in duplicate and >100 cells have been imaged per condition. In **b**) a quantification of lysosomes per cell for 30 randomly chosen cells per condition is shown. Symbols represent single cell data; black lines represent mean +/- s.d.

Proteins are surrounded by hydration layers similar to those of lipid bilayer membranes. If these were impaired by ice formation, it could lead to denaturing of cellular proteins. E.g. a strong denaturation of overall secondary protein structure was shown after holding cells for 60 min at -80°C following comparably slow cooling [9]. I compared therefore structural integrity of cytosolic proteins from HeLa cells frozen and thawed rapidly in OPS by circular dichroism spectroscopy. We could not detect any denaturing in protein extracts even after rapid freezing and thawing in OPS without cryoprotectants (Figure A in S1 Fig), which is lethal to the cells (Huebinger et al. 2016; Fig 2B). It is still possible that there was specific damage to a small subset of cytosolic proteins, which would not be detectable in this bulk measurement, or to membrane proteins, which were not measured here. However, these results indicate that cryodamage under rapid freezing and thawing conditions is not associated with extensive protein denaturation, as it was observed by others upon slower cooling [9]. Even though a majority of cells had intact plasma membranes when pre-treated with 10 mM cholesterol or 5mM ergosterol, they did not re-adhere after rewarming (Fig 2B and 2D). This indicates that they still get lethal damage. Since lysosomes are one of the organelles that have very low endogenous cholesterol content and are hardly reached by exogenous cholesterol [13], we checked their integrity in cells treated with cholesterol-loaded MβCD after non-equilibrium cryopreservation. Lysosome integrity was measured by fluorescence microscopy of the dye acridine orange, which gets trapped in lysosomes due to their low pH. Upon concentration increase in these compartments it changes from green to red fluorescence [15]. By this method, 15 +/- 5 (mean +/- s.d.) lysosomes per HeLa cells were detectable as red dots in normal suspended cells. After rapid freezing and thawing without cryoprotectants, these dots were absent from HeLa cells with normal sterol content. This indicates that lysosomes got ruptured upon freezing. Enrichment with cholesterol before cooling did not protect the lysosomes from this damage. 90% of these cells showed two or less lysosomes and the remaining 10% had also an abnormal low number of lysosomes (Fig 3). This shows that intracellular organelles like lysosomes still got ruptured upon rapid cryopreservation, which explains why the cells cannot survive. Most cells frozen in DE-medium showed a normal number of intact lysosomes. Only 23% of these cells showed two or less lysosomes (Fig 3). This agrees well with the observed 80% survival rate of HeLa cells after rapid cooling and thawing in this medium [6].

## Discussion

It has been assumed for a long time that the success of cryopreservation depends on the avoidance of intracellular ice crystals [1,5]. However, we found previously that rapidly-cooled cells can actually tolerate intracellular ice crystals [6]. The results presented here show that under these non-equilibrium cooling conditions cellular membranes are a major target for cryodamage. A massive denaturing of proteins, as it was observed upon relatively slow lethal cooling [9], was not observed under lethal rapid cooling conditions. Importantly, the damage to plasma membranes can be suppressed by solely changing their properties, without interfering with ice formation. This shows that there is a cryoprotective effect that is independent of the suppression of ice formation. In this context, it is of note that the mechanism of cryoprotection is specific for rapid cooling approaches, which prevent the samples from reaching thermodynamic equilibrium during cooling. Upon slow cooling, membrane integrity was actually adversely affected by sterol-loaded MβCD (Fig 2D) and led to strong and irreversible denaturing of proteins [9]. This indicates that there is considerable difference in the mechanism of cryodamage between equilibrium (i.e. slow-cooling) and non-equilibrium cooling processes.

To my knowledge, a cryoprotective effect of sterols has not been described before in somatic cells. It has however been observed empirically that there is a tendency of higher sperm motility (0–20% depending on the species) after cryopreservation with increased cholesterol content in presence of other cryoprotectants [16]. Likewise, *in vitro* fertilized bovine oocytes had a higher cleavage rate and developed more eight-cell embryos after cryopreservation with cholesterol [17]. The positive effect of enhanced cholesterol in sperm cells has been assigned to a prevention of cold shock damage above 0°C in sperm cells with low cholesterol content [10]. For somatic cells, such cold shock is normally not observed and cryodamage occurs at lower temperatures in the presence of ice [1], which is also the case in HeLa cells [6]. Cold shock damage in sperm cells is associated with a phase change in the membrane [10]. It is possible that this membrane phase change just co-occurs with recrystallization in somatic cells and is responsible for the cryo-damage. It has indeed been shown that (upon slow cooling) phase changes of membranes in somatic cells actually occur in a temperature regime, where ice is present and that cryoprotective agents like DMSO can modulate this phase change [8]. Phase changes of cellular membranes can be rather complex due to the many constituents of the membranes, which also differ considerably in composition among cell types [10,13,18,19]. Further, rapid cooling should also lead to different membrane configurations than slow cooling, since it does not allow the membranes to reach their thermodynamic equilibrium state. For these reasons, not much is known about the state of membranes after rapid cooling conditions. However, the membrane damage in somatic cells could also be of different nature and be directly or indirectly caused by recrystallization. In analogy to slow cooling, high solute concentration upon ice formation could be the reason [1,7]. However, this would imply an additional denaturing of proteins, as seen by others upon slow cooling [9]. Yet, this was not detectable. Moreover, the highest amount of cryodamage after rapid cooling appeared at relatively high subzero temperature, at which the total amount of ice and therefore also the concentration of solutes in the unfrozen fraction is relatively low [6]. This indicates that the solute concentration is not the major source of membrane damage upon rapid cooling/rewarming. On the other hand, it has been argued that a model of ice crystals piercing lipid-bilayer membranes is unlikely on a molecular level, since ice-crystals form at their growing edge and have no driving force [20]. Additionally, intracellular ice crystals appear under these conditions not as sharp-edged entities, but rather spherical [6]. Yet, since the density of ice is lower than that of liquid (or vitrified) water, intracellular ice crystals could become too big for the cellular organelles or even the whole cell upon recrystallization, which could lead to their bursting. For these reasons, it is not clear yet, if membrane damage is caused by recrystallization or just co-occurring with it. Our results however show that increasing the cholesterol content and treating the cells with empirically found cryoprotective agents both lead to formation of a phase separation into a fast moving mobile and a slow moving/immobile phase in the plasma membrane of HeLa cells before freezing (Fig 1). How exactly these phases are composed and if and how this primes the membranes for non-equilibrium cryopreservation will need further investigations. It would be however very interesting to identify methods to modulate also intracellular membranes in a similar way without the additional effects of DMSO or ethylene glycol.

It is the scope of this publication to demonstrate that modulation of membrane properties can protect cells upon cryopreservation, not to present cholesterol as ready-made, self-sufficient cryoprotective agent for somatic cells. Cholesterol treatment did actually not lead to survival of HeLa cells and lysosomes in the cells got ruptured even in cells treated with cholesterol. This is likely caused by the tight regulation of intracellular cholesterol in somatic cells, where most of the cholesterol (e.g. >90% in human fibroblasts) remains in the plasma membrane and e.g. lysosomes are hardly reached even by exogenous cholesterol [13,18]. The distribution of cholesterol can be very different in sperm cells. Several types of organelles such as endoplasmic reticulum, golgi apparatus or lysosomes are not found in sperm cells. However, they possess specialized organelles such as the acrosome, which can have a rather high cholesterol content. E.g. in bovine sperm the cholesterol content was measured to be 21% [19]. In these cells, only 11% of total cholesterol was found in the plasma membrane [19]. In contrast to somatic cells, the plasma membrane of these cells might thus be their weakest membrane in terms of cryo-resistence. This might explain, why the survival after cryopreservation of these cells can be efficiently increased by external application of cholesterol (in addition to classical cryoprotective agents) [16].

I believe that identifying cellular membranes as primary targets for cryodamage upon rapid cooling and thawing and demonstrating the possibility to protect them in the presence of ice and absence of classical cryoprotectants adds a very important new facet to the complete understanding of mechanisms of cryoprotection. Classical cryoprotectants are so far found empirically. It has been pointed out in a very recent review that this did not lead to discovery of new classes of cryoprotective agents since Ashwood-Smith compiled a list in 1987 [7]. Hence, a thorough understanding of the underlying mechanisms is necessary to allow for the intelligent design of new types of cryoprotective agents that are more efficient and less cytotoxic.

## Materials and methods

### Cell culture

HeLa (ATCC No. CCL-185) were cultured in Dulbecco's Modified Eagle Medium (DMEM) supplemented with 10% fetal bovine serum (FBS), 100 μg/mL streptomycin, 100 U/mL penicillin, 1% L-Glutamine (200mM), and 1% nonessential amino acids (all PAN-Biotech, Aidenbach, Germany). HeLa Cells were authenticated by Short Tandem Repeat (STR) analysis and did not contain DNA sequences from mouse, rat and hamster (Leibniz Institute DSMZ). Cells were regularly tested for mycoplasma infection using the MycoAlert Mycoplasma detection kit (Lonza, Basel, Switzerland) and cultured at 37°C with 95% air and 5% CO_2_.

### Composition of cryoprotective DE-medium

DE-medium has been prepared as used before [6]. 15% DMSO, 15% ethylene glycol (Serva, Heidelberg, Germany) were mixed in phosphate buffered saline (PBS) containing 20% FCS. The medium has been termed DE-medium as abbreviation for its main cryoprotective substances DMSO and ethylene glycol. HeLa cells have been exposed directly to this medium, this results in a transient shrinkage of these cells of about 30–60 s [6]. Measurements have been started 5 min after application of the medium, to be sure that the medium was completely equilibrated. The survival of HeLa cells after 5 min incubation in DE medium was measured by their ability to re-adhere after detachment to be 95 +/- 4% (mean +/- s.d.; n = 5). Survival after freezing and thawing in OPS was on average 80% [6].

### Modification of sterol content by methyl-β-cyclodextrin (MβCD)

MβCD is a cyclic oligosaccharide. Due to its hydrophobic core, it can transport lipids to and from lipid bilayer membranes. Due to the size of this core, it is most efficient in transporting sterols [21]. When MβCD is pre-loaded with a sterol it delivers the sterols to lipid bilayer membranes. For delivery of cholesterol or ergosterol to membranes they have been pre-loaded by dissolving 80 mg/mL MβCD (Sigma-Aldrich GmbH, Taufkirchen, Germany) in PBS and adding 6 mM of cholesterol or ergosterol (Sigma-Aldrich GmbH, Taufkirchen, Germany). The solution was shaken over night at 30°C and filtered through a 0.2 μm filter (Merck Millipore, Billerica, MA, USA). The solution was diluted to final concentrations of MβCD, as indicated in the corresponding figures, in HEPES-buffered DMEM without phenol red or PBS before adding it to the cells.

### Quantification of sterols

A solution of 10^6^ HeLa cells in 200 μL of HEPES-buffered DMEM with or without sterol-loaded MβCD was centrifuged in a table-top centrifuge to pellet the cells (135 x g; 2 min). To extract the lipids an established protocol was used with slight modifications [22]. The supernatant was removed and 200 μL of ice-cold methanol were added. The following steps were performed on ice. Samples were vortexed and incubated for 10 min. 400 μL chloroform were added; the samples were vortexed and incubated for 5 min in an ultrasonic bath. 125 μL of 0.88% KCl in water were added, vortexed and incubated for 10 min. They were centrifuged at 200 x g for 5 min. The upper phase was removed and 300 μL of the lower phase was transferred to a fresh tube. The lipids were dried in a vacuum concentrator (Concentrator plus 5305, Eppendorf AG, Hamburg, Germany) and subsequently re-suspended in 50 μL heptane.

5 μL of the lipid solution were used to quantify cholesterol fluorometrically using the Amplex^TM^ Red cholesterol assay kit (Thermo Fisher Scientific, Waltham, MA, USA) following the protocol of the manufacturer. Briefly, different dilutions of a cholesterol reference standard, a positive control with 20 mM H_2_O_2_ and the test samples were diluted in reaction buffer to a final volume of 50 μL and pipetted into separate wells of a 96-well plate. 50 μL of a solution of 300 μM Amplex^TM^ Red reagent, 2 U/mL horseradish peroxidase, 2 U/mL cholesterol oxidase, 0.2 U/mL cholesterol esterase were added per well. After > 30 min incubation in the dark, the fluorescence was measured with excitation at 560 nm and emission at 590 nm in a microplate reader (SpectraMax M5, Molecular Devices LLC, San Jose, CA, USA). Cholesterol in the test samples was quantified using a linear fit through the different dilutions of the reference standards.

20 μL of the lipid solution plus 50 μL heptane solution were used to measure the ergosterol concentration. Absorption spectra of this solution were recorded from 220 nm to 310 nm using a DU800 spectrometer (Beckmann Coulter, Brea, CA, USA). The basal level was normalized to the adsorption at 310 nm. A spectrum of a control sample without ergosterol was subtracted. Using these spectra, a linear correlation of the peaks at 270 nm, 280 nm and 292 nm with ergosterol concentration was obtained using an ergosterol reference standard. A linear fit for the absorption at 270 nm of the reference standard was used to quantify the ergosterol concentration in test samples.

### Fluorescence recovery after photobleaching (FRAP)

For FRAP experiments HeLa cells have been seeded in glass bottom dishes (MatTek Corporation, Ashland, MA, USA) with 2 x 10^5^ cells per dish the day before the experiment. For cholesterol enrichment, cells have been treated with 10 mM cholesterol loaded MβCD in HEPES-buffered cell culture medium for 60 min and subsequently washed with phosphate buffered solution. The solution has been removed completely and 100 μL of DiOC18 in phosphate buffered solution has been added (Vybrant-DiO 1:200, Thermo Fisher Scientific, Waltham, MA, USA). After 15 min of incubation at 37°C, cells have been washed three times with PBS. FRAP measurements were performed on a Leica SP5 confocal microscope using a 60x 1.4 NA oil immersion objective (Leica Microsystems CMS GmbH, Mannheim, Germany). The incubation chamber around the microscope was set to 37°C. Imaging was performed with the 488-nm laser line of an argon laser set to 70% laser power with less than 10% transmission. Images were acquired at a rate of 1.318 s/image. 5 frames were recorded before bleaching and 72 after bleaching. Bleaching was performed by zooming in on the bleaching area and scanning once with all lines of the argon laser at 100% transmission plus an UV diode at 100% transmission. This resulted in bleaching of > 80% of the fluorescence intensity in the bleached spot. For analysis, the background fluorescence outside of the cells was first subtracted from the image. The intensity in the bleached spot was normalized to the intensity in the whole cell to correct for the total loss of fluorescence during the experiment. It was further normalized to pre-bleaching intensity in the spot. The obtained fluorescence recovery curves were fitted with a single exponential fit using the software Origin (OriginLab Corporation, Northampton, MA, USA). The mobile fraction (mf) was calculated using the asymptote (y0) of this function:

$$mf=(y0-I_{bleached}/I_{prebleached}-I_{bleached})$$

Where I_bleached_ is the fluorescence intensity in the bleached area in the first frame after bleaching and I_prebleached_ is the mean intensity in that area in the 5 frames before bleaching. Accordingly, the immobile fraction (if) is:

if=1−mf

The diffusion constant (D) was calculated using the time to reach half maximum (τ_1/2_) of the exponential fit and the equation for two-dimensional diffusion [23]:

$$D=r^{2}/{4\tau }_{1/2}$$

with r as the radius of the bleached area.

### Freezing and thawing in open pulled straws (OPS)

HeLa cells were frozen in a concentration of 5 x 10^6^ cells/mL. There are methods to cool and warm samples more rapidly than in OPS and achieve higher survival rates [24]. However, it was not the objective to optimize the cooling protocol, but to investigate the mechanism of cryoprotection. For this, a suboptimal cooling performance, which can however still be considered a non-equilibrium process, is actually beneficial. To load the OPS (Vajta embryology consulting, RVT, Australia), a small drop (2 μL) of cell suspension was placed on a petri dish. The drop was taken up into the OPS by capillary forces. For rapid cooling, the device was held parallel above the liquid nitrogen surface before the tip was rotated into the liquid nitrogen. This technique was shown to foster faster cooling than just dipping the device in the liquid nitrogen [25]. The solution in the tip of the OPS was warmed in a 0.5 μL tube (Eppendorf AG, Hamburg, Germany) containing 200 μL cell culture medium at 37°C to ensure fast and uniform warming by immersing the whole cell suspension into the medium.

### Freezing of cells with 1°C/min

To freeze cells slowly with 1°C/min, HeLa cells were suspended with 2x10^6^ cells per mL in HEPES-buffered cell culture medium with or without cholesterol-loaded MβCD. 500 μL cell suspension was placed in 2mL-cryovials and incubated for 1 h at 37°C. The cryovials were then placed in a Mr. Frosty^TM^ freezing container (Thermo Fisher Scientific, Waltham, MA, USA), which was placed into freezer at -80°C. By this samples are cooled at rates close to 1°C/min. After 6–7 h, samples were taken out of the Mr. Frosty^TM^ device and transferred to a freezer at -150°C. Samples were thawed by transferring them directly and rapidly to a water bath at 37°C.

### Test for plasma membrane integrity by propidium iodide (PI) staining

HeLa cells were stained with 12 μg/mL PI solution in phosphate buffered solution. Cells frozen in OPS were directly thawed in 200 μL of this solution at 37°C. Suspensions of control cells and slow-frozen cells were diluted 1:100 in this solution. Transmitted light and red fluorescence images were recorded at 20 randomly chosen positions at a Zeiss Axiovert microscope using a 40x 1.3 NA oil immersion objective. The total number of cells was deduced from the transmitted light images. Cells were counted as PI-positive, if red fluorescence was visible in the nucleus.

### Test for cell survival

The viability of HeLa cells after cooling and rewarming was quantified by assessing the proportion of cells that were able to re-adhere. This method can be used to quantify overall cellular survival, not just direct membrane ruptures [24]. After treatment, cells were seeded in cell culture dishes and cultured at 37°C and 5% CO_2_. Control cells always adhered within three hours. Cells not adhering within three hours after re-seeding did also not adhere later on, as observed microscopically. Because of this observation, adherence was always quantified after six to seven hours. Therefore, the supernatant was removed and cell concentration was determined using a hemocytometer. This reflects the fraction of dead cells not able to re-adhere. Adherent cells were washed once in PBS, detached by trypsinization and taken up into a solution of fresh medium. The concentration of these living cells was also determined using a hemocytometer.

### Circular dichroism (CD) spectroscopy

HeLa cells have been seeded with 2x10^6^ cells per 10 cm petri dish the day before. Cells were washed once with phosphate buffer. For native protein extraction, they were harvested by scraping on ice in a 500 μL buffer per petri dish. The buffer consisted of 150 mM NaCl, 1 mM EDTA-Na_2_, 20 mM Tris-Cl, 1 mM EGTA, 2.5 mM Tetrasodiumpyrophosphat, 0.1% Igepal, 10 mM PMSF (all Sigma-Aldrich GmbH, Taufkirchen, Germany) and 1 tablet cOmplete Mini EDTA-free protease inhibitor (Roche Diagnostics GmbH, Mannheim, Germany). They were transferred to a 15-mL falcon tube and kept on ice. Cells were lysed on ice by sonication three times for 12 s at 40% power using a Sonopuls HD 2200 (Bandelin electronic GmbH, Berlin, Germany). They were centrifuged at 3000 x g and 4°C for 20 min and the supernatant was collected for CD-spectroscopy. Protein concentration in the solution was determined using the bicinchoninic acid assay using the Micro-BCA^TM^ Kit (Thermo Fisher Scientific, Waltham, MA, USA). CD-spectra were recorded using a J-810 spectrometer (Jasco Deutschland GmbH, Pfungstadt, Germany).

### Imaging of lysosomes with acridine orange staining

5x10^5^ HeLa cells were seeded per 3.5 cm petri dish one day before the experiment. The cell culture medium was removed and they were stained with 1 mL 0.04 mg/mL acridine orange in PBS for 15 min at 37°C. After washing with PBS, cells were trypsinized with 100 μL Trypsin solution. When all cells were detached, the reaction was stopped by addition of 900 μL DMEM containing 10% FBS. The solution was transferred to a 1.5 mL tube and spun down in a table-top centrifuge (150 x g; 5 min). The supernatant has been removed and cells have been taken up in 200 μL medium (HEPES-buffered DMEM +/- MβCD or DE-medium as indicated in the corresponding figure). After the treatments indicated in the experiments, 2 μL of this solution has been diluted with 200 μL cell culture medium in 8-well-Lab-Tek^TM^ glass bottom dishes for microscopy. The cells have been imaged on a Leica SP5 confocal microscope with a 60x 1.4 NA oil immersion objective (Leica Microsystems CMS GmbH, Mannheim, Germany). The sample was excited by the 488-nm laserline of an argon laser. Green fluorescence was collected at 500–560 nm and red fluorescence at 600–700 nm.

## Supporting information

S1 Fig CD-spectroscopy of cytosolic proteins from HeLa cells. Shown are circular dichroism (CD) spectra from 200 nm to 260 nm. CD-spectra in this region are characteristic for secondary structural elements (α-helices and β-sheets). Upon denaturing of the proteins, the amplitude of CD spectra decreases in this region. **a)** Cytosolic protein extracts from HeLa cells have been frozen in OPS without cryoprotective agents in two different concentrations and measured in a CD spectrometer (50 mg/mL red lines; 3 mg/mL green lines). The protein extracts have been also measured without freezing (black and grey lines). **b)** Cytosolic protein extracts from HeLa cells have been heat denatured at 40°C, 50°C and 90°C.
(EPS)
